# Global, regional and national burden of orofacial clefts from 1990 to 2021: Analysis of risk factors and prediction of trends in 2035

**DOI:** 10.1097/MD.0000000000049701

**Published:** 2026-07-17

**Authors:** Jiangbo Xin, Haoxuan Sun, Weiling Chen

**Affiliations:** a Department of Oral and Maxillofacial Surgery, Hebei Eye Hospital, Xingtai, China.

**Keywords:** ARIMA, disability-adjusted life years, Global Burden of Disease, health disparities, orofacial clefts, Socio-demographic Index

## Abstract

Orofacial clefts (OFCs) are common congenital anomalies with substantial health and social consequences, particularly where access to surgery and longitudinal care is limited. Burden varies across regions and socioeconomic strata. Using Global Burden of Disease 2021 estimates, we analyzed OFC incidence, prevalence, mortality, years of life lost, years lived with disability, and disability-adjusted life years (DALYs) from 1990 to 2021 at global, regional, and national levels. Analyses were stratified by Socio-demographic Index (SDI) quintiles and World Bank regions. We applied autoregressive integrated moving average models to project prevalence and DALY trends to 2035. In 2021, OFCs affected an estimated 4.12 million individuals worldwide, with an age-standardized DALY rate of 5.18 per 100,000 population. The highest burdens were observed in Oceania, Western sub-Saharan Africa, and South Asia – regions predominantly in the lower SDI strata. Projections indicate gradual global reductions in prevalence and DALY rates through 2035; however, declines are expected to proceed more slowly in high-SDI regions than in low-SDI regions. OFCs remain a notable global health burden with marked geographic and socioeconomic disparities. Priorities include expanding access to timely surgical repair and multidisciplinary follow-up, strengthening public health outreach, and embedding psychosocial support – particularly in low-SDI settings.

## 1. Introduction

Orofacial clefts (OFC) are among the most common congenital structural defects in humans, caused by malformations of the upper lip, primary palate, secondary palate, and other orofacial structures during craniofacial development.^[1,2]^ Global prevalence is estimated at roughly 1 per 700 live births, with marked heterogeneity by geography, ancestry, and socioeconomic status.^[3]^ Phenotypically, OFCs are classically partitioned into cleft lip with or without cleft palate (CL/P) and isolated cleft palate (CP/CPO).^[4]^ Clinically, cleft lip refers to a unilateral or bilateral gap between the philtrum and the lateral upper lip, usually extending through the upper lip and maxilla to the nostril, sometimes involving a secondary cleft palate; cleft palate refers to an isolated defect in the secondary palate while the upper lip remains intact.^[5,6]^ OFC can be classified into 5 types based on different combinations of cleft lip and cleft palate: unilateral cleft lip, bilateral cleft lip, unilateral cleft lip with cleft palate, bilateral cleft lip with cleft palate, and isolated cleft palate.^[7]^ The global prevalence of cleft lip and palate is 1.82 per thousand live births, and in some regions, cleft lip and palate have become one of the top 2 birth defects.^[8]^ Although surgical interventions can alleviate feeding difficulties, speech impairment, and breathing problems associated with cleft lip and palate, the resulting psychological issues and economic burden are often unaffordable for most patients. Therefore, investigating the pathogenesis of cleft lip and palate is not only important for understanding the complex regulation of biological development but also provides a theoretical basis for preventing the occurrence of OFC. However, barriers to timely surgical intervention result in a substantial backlog of untreated cases globally, contributing to a high disease burden measured in disability-adjusted life years (DALYs).^[9]^

The Global Burden of Disease (GBD) 2021 study provides comparable estimates of prevalence, DALYs, years lived with disability, and years of life lost for OFCs across countries and over time. These data consistently show higher burden in regions with lower Socio-demographic Index (SDI) scores, underscoring the influence of structural socioeconomic determinants on outcomes of congenital anomalies.

Addressing the global burden of OFCs requires a multifaceted approach that includes expanding access to surgical repair and integrating comprehensive psychosocial support, particularly in underserved regions. Strengthening international collaboration, investing in healthcare infrastructure, and promoting awareness about OFCs can significantly reduce the global burden and improve the quality of life for affected individuals. This study, based on GBD 2021 data, aims to provide insights into the epidemiology of OFCs and their associated burden, ultimately guiding future initiatives to mitigate health disparities observed across different socioeconomic settings.

## 2. Materials and methods

### 2.1. Overview and definition of GBD 2021

GBD 2021 provides harmonized estimates for 371 conditions across 204 countries and territories, including incidence, prevalence, mortality, and risk factors.^[10,11]^ Data are compiled from censuses, household surveys, civil registration and vital statistics, disease registries, case notifications, health service utilization data, air-pollution monitoring, satellite imagery, and other repositories.^[12]^ Each type of data is identified from systematic reviews of published studies, searches of government and international organization websites, published reports, demographic and health surveys, and data sets provided by GBD collaborators.^[12]^ Every newly identified data source is uniquely tagged by a designated team of librarians and integrated into the Global Health Data Exchange (GHDx). GHDx ensures public access to metadata and actual data from each source in GBD, provided data provider approval is obtained. The GHDx source tool also allows users to pinpoint specific datasets used to estimate outcomes related to any specific disease or injury in different settings. Peer-reviewed publications^[11,13]^ provide extensive literature detailing the data collection and statistical modeling methods used in GBD 2021. In the GBD 2021 framework, OFCs are defined as congenital malformations of the lip and palate and correspond to ICD-10 codes Q35–Q37. In this specific study, data related to LOC and its associated attributable risk factors from 1990 to 2021 were analyzed using the GHDx query tool. Because the GBD study data can be downloaded from an open access database, ethics approval and informed consent were not required for this study.

### 2.2. Measurement of disease burden

This study investigated 6 indicators – incidence, mortality, prevalence, DALY, years lived with disability, and years of life lost – to elucidate the disease burden associated with LOC. The definitions and calculation methods for these indicators have been extensively elaborated in previous academic literature.^[11–14]^ In our study, we provided detailed descriptions of the point estimates and their respective 95% uncertainty intervals (UI). These intervals were calculated based on 1000 replicate dataset samples, with the upper and lower bounds determined using the 2.5th and 97.5th percentiles of the uncertainty distribution. Additionally, age-standardized estimates were derived based on the global age structure in 2021.^[15]^

### 2.3. SDI analysis

The GBD 2021 study used the SDI as an indicator of the economic and social conditions that influence health outcomes in different regions. SDI represents the geometric mean of a 0 to 1 index of the total fertility rate for individuals under 25 years, the average educational attainment for those aged 15 and older, and lag-distributed income per capita.^[5]^

### 2.4. Geographical distribution

We summarized global, regional, and national OFC burden and prevalence, identified highest-burden countries in 2021, and contrasted patterns across SDI and World Bank regional groupings.

### 2.5. Statistical analysis

Data analysis and visualization were generated using R statistical software (version 4.3.3). Based on data from 1990 to 2021, the autoregressive integrated moving average (ARIMA) model is used to forecast the DALY rate and prevalence rate for the period 2022 to 2035. ARIMA is a popular statistical model used for time series forecasting. It is particularly effective for univariate time series data, where future values depend on past values.

### 2.6. STROCSS criteria were followed in this work

This study has been conducted and reported following the STROCSS^[11]^ (Strengthening the Reporting of Cohort Studies in Surgery) criteria, ensuring comprehensive and transparent reporting of observational cross-sectional studies.

## 3. Results

In 2021, the global prevalence of OFC across all genders and age groups was 4.12 million (95% UI, 3.32–5.03 million), with 434,300 DALYs (95% UI, 265,600–735,000).

### 3.1. Global burden

The estimated global DALY rate is 5.18 per 100,000 population (95% UI: 3.2 to 8.5 DALYs; Table 1). The highest burden of OFC is in Oceania (24.75 DALYs per 100,000; 95% UI: 6.17 to 60.6 DALYs per 100,000), followed by Western sub-SaharanAfrica (10.52 DALYs per 100,000; 95% UI: 3.72 to 37.67 DALYs per 100,000) and North Africa and the Middle East (9.39 DALYs per 100,000; 95% UI: 5.6 to 20.08 DALYs per 100,000). The lowest burden of OFC is in high-income North America (1.2 DALYs per 100,000; 95% UI: 0.72 to 1.86 DALYs per 100,000), followed by Western Europe (1.56 DALYs per 100,000; 95% UI: 0.96 to 2.36 DALYs per 100,000) and Southern Latin America (1.65 DALYs per 100,000; 95% UI: 1.05 to 2.53 DALYs per 100,000; Table 2).

**Table 1:** Disability-adjusted life years (DALYs) and prevalence of orofacial clefts across different SDI regions in 2021.

Location	DALYs	Rate (per 100,000)	Prevalence	Rate (per 100,000)
Global	408,775.34 (252,320.08–671,119.88)	5.18 (3.2–8.5)	4,124,006.77 (3,318,692.55–5,026,199.55)	52.26 (42.05–63.69)
Low SDI	126,685.11 (49,746.3–344,500.56)	11.34 (4.45–30.83)	722,909 (583,611.35–879,494.83)	64.7 (52.23–78.71)
Low-middle SDI	134,879.52 (88,416.21–201,658.23)	7.02 (4.6–10.5)	1,470,278.25 (1,169,396.49–1,805,705.62)	76.53 (60.87–93.99)
Middle SDI	92,289.21 (65,013.78–12,9542.5)	3.77 (2.66–5.29)	1,148,111.39 (927,870.18–1,400,797.01)	46.89 (37.89–57.21)
High-middle SDI	32,495.73 (21,979.16–48,008.91)	2.49 (1.69–3.68)	436,311.09 (356,149.09–530,335.51)	33.46 (27.31–40.67)
High SDI	22,030.85 (13,635.59–33,496.89)	2.01 (1.25–3.06)	343,810.24 (275,876.35–419,985.22)	31.43 (25.22–38.39)

DALYs = disability-adjusted life years, SDI = Socio-demographic Index.

**Table 2:** Disability-adjusted life years (DALYs) and prevalence of orofacial clefts across different regions in 2021.

Location	DALYs	Rate (per 100,000)	Prevalence	Rate (per 100,000)
Central Asia	5597.13 (3769.18–8351.78)	5.84 (3.93–8.72)	76,835.2 (62,046.74–92,647.68)	80.2 (64.76–96.7)
East Asia	36,372.04 (24,862.63–52,967.48)	2.47 (1.69–3.6)	429,540.63 (347,839.7–519,201.44)	29.17 (23.62–35.25)
High-income Asia Pacific	5517.05 (3380.13–8453.58)	2.98 (1.82–4.56)	90,815.22 (71,751.15–111,736.41)	48.97 (38.69–60.25)
South Asia	122,621.63 (76,631.14–193,338.08)	6.64 (4.15–10.47)	1,633,290.62 (1,295,458.2–2,022,505.8)	88.45 (70.15–109.53)
Southeast Asia	35,296.75 (23,249.23–53,115.92)	5.05 (3.33–7.61)	294,296.49 (238,649.69–359,900.26)	42.14 (34.18–51.54)
Australasia	550.87 (348.75–839.18)	1.78 (1.13–2.71)	8317.18 (6674.3–10,154.63)	26.86 (21.56–32.8)
Oceania	3446.46 (859.03–8439.71)	24.75 (6.17–60.6)	4786.16 (3804.61–5947.68)	34.36 (27.32–42.7)
Central Europe	1941.03 (1198.84–2954.31)	1.68 (1.04–2.56)	30,463.75 (24,477.34–36,896.12)	26.43 (21.24–32.01)
Eastern Europe	3537.87 (2200.81–5391.74)	1.71 (1.06–2.61)	52,312.99 (41,814.62–64,112.68)	25.3 (20.22–31.01)
Western Europe	6841.68 (4201.06–10,342.15)	1.56 (0.96–2.36)	106,619.54 (86,208.23–128,179.74)	24.38 (19.71–29.31)
Andean Latin America	3210.56 (2335.84–4508.42)	4.85 (3.53–6.82)	33,534.43 (27,121.25–40,758.96)	50.71 (41.01–61.63)
Central Latin America	9694.69 (6999.83–13,400.69)	3.83 (2.77–5.3)	96,756.99 (78,596.34–117,245.66)	38.24 (31.07–46.34)
High-income North America	4438.08 (2676.18–6902.86)	1.2 (0.72–1.86)	68,252.28 (52,114.67–86,705.82)	18.44 (14.08–23.42)
Southern Latin America	1114.12 (711.78–1711.85)	1.65 (1.05–2.53)	16,307.06 (12,730.29–19,984.63)	24.09 (18.81–29.52)
Tropical Latin America	4984.85 (3585.14–7008.7)	2.19 (1.58–3.08)	53,196.57 (43,518.49–63,293.21)	23.38 (19.13–27.82)
Caribbean	2049.25 (1218–3328.95)	4.32 (2.57–7.01)	21,561.25 (17,052.46–26,691.96)	45.43 (35.93–56.24)
North Africa and Middle East	58,524.48 (34,890.17–125,071.93)	9.39 (5.6–20.08)	532,165.86 (425,774.6–646,873.5)	85.42 (68.34–103.83)
Central sub-Saharan Africa	8642.34 (3613.95–24,278.77)	6.31 (2.64–17.73)	55,173.75 (44,008.9–68,507.1)	40.29 (32.14–50.03)
Eastern sub-Saharan Africa	36,756.97 (14,299.28–113,593.01)	8.63 (3.36–26.66)	221,976.32 (179,210.67–271,859.51)	52.1 (42.06–63.8)
Southern sub-Saharan Africa	6097.99 (3886.31–8970.25)	7.59 (4.84–11.17)	46,724.69 (37,840.33–57,921.63)	58.18 (47.12–72.13)
Western sub-Saharan Africa	51,539.49 (18,210.08–184,528.48)	10.52 (3.72–37.67)	251,079.81 (202,129.27–306,333.58)	51.26 (41.27–62.54)

DALYs = disability-adjusted life years.

The disease burden is negatively correlated with SDI. Low SDI countries reported the highest burden of OFC (11.34 DALYs per 100,000; 95% UI: 4.45 to 30.83 DALYs per 100,000), followed by low-middle SDI countries (7.02 DALYs per 100,000; 95% UI: 4.6 to 10.5 DALYs per 100,000), middle SDI countries (3.77 DALYs per 100,000; 95% UI: 2.66 to 5.29 DALYs per 100,000), high-middle SDI countries (2.49 DALYs per 100,000; 95% UI: 1.69 to 3.68 DALYs per 100,000), and high SDI countries (2.01 DALYs per 100,000; 95% UI: 1.25 to 3.06 DALYs per 100,000).

### 3.2. Global prevalent rate

Globally, 52.26 per 100,000 people are affected by OFC (95% UI: 42.05 to 63.69 per 100,000; Table 1). The highest prevalence of OFC is in South Asia (88.45 per 100,000; 95% UI: 70.15 to 109.53 per 100,000), followed by North Africa and the Middle East (85.42 per 100,000; 95% UI: 68.34 to 103.83 per 100,000), and Central Asia (80.2 per 100,000; 95% UI: 64.76 to 96.7 per 100,000). The lowest prevalence is in high-income North America (18.44 per 100,000; 95% UI: 14.08 to 23.42 per 100,000), Tropical Latin America (23.38 per 100,000; 95% UI: 19.13 to 27.82 per 100,000), and Southern Latin America (24.09 per 100,000; 95% UI: 18.81 to 29.52 per 100,000; Table 2).

By SDI, the highest prevalence of OFC is in low-middle SDI countries (76.53 per 100,000; 95% UI: 60.87 to 93.99 per 100,000), followed by low SDI countries (64.7 per 100,000; 95% UI: 52.23 to 78.71 per 100,000), middle SDI countries (46.89 per 100,000; 95% UI: 37.89 to 57.21 per 100,000), and high-middle SDI countries (33.46 per 100,000; 95% UI: 27.31 to 40.67 per 100,000). The lowest prevalence is in high SDI countries (31.43 per 100,000; 95% UI: 25.22 to 38.39 per 100,000).

### 3.3. Country-specific burden and prevalent rate

**Figure 1.**
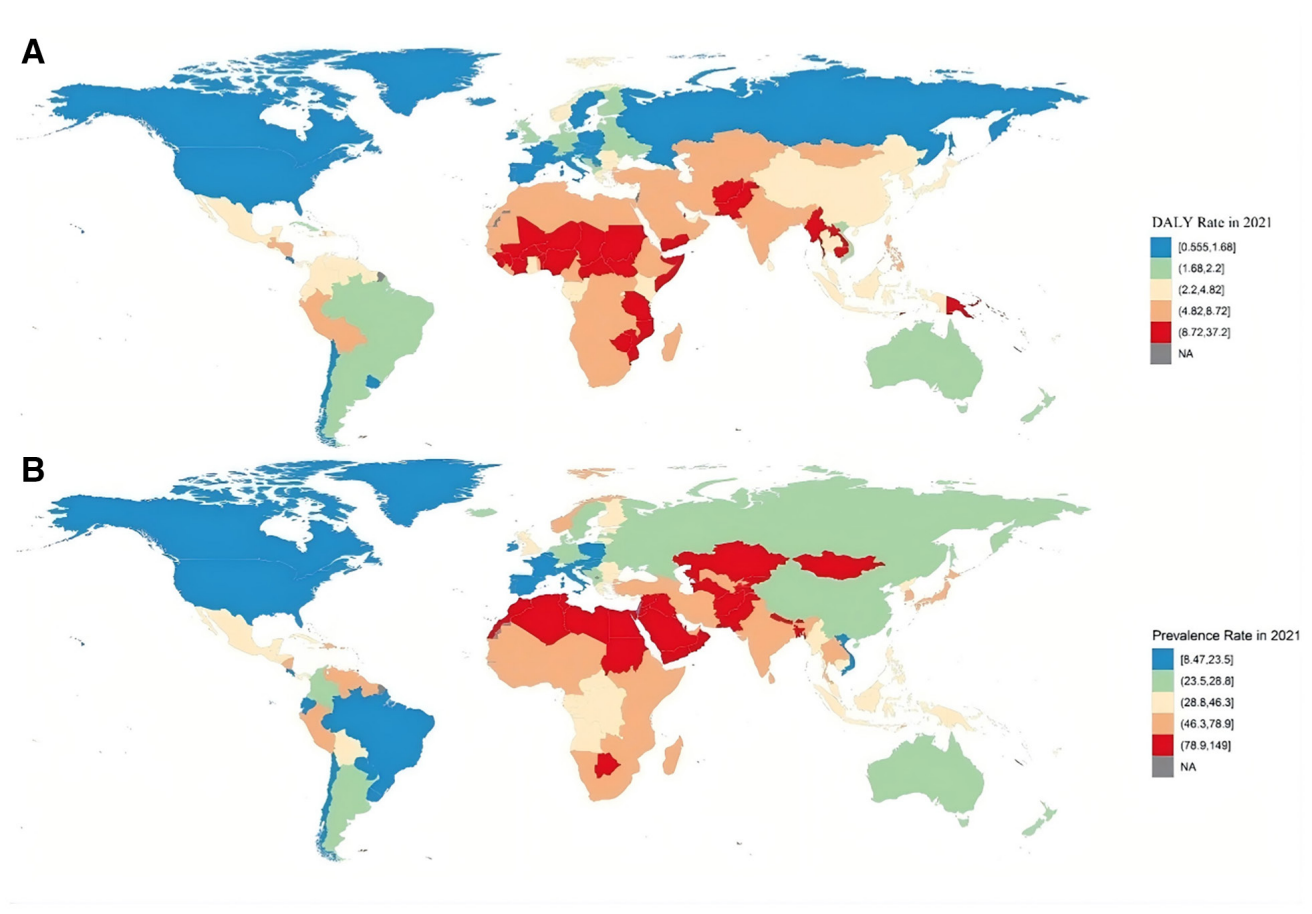
Disability-adjusted life years (DALYs) rate (A) and prevalence rate (B; per 100,000 population) of orofacial clefts across countries and territories in 2021.

The highest burden of OFC is in Afghanistan (37.21 DALYs per 100,000; 95% UI: 7.14 to 186.55 DALYs per 100,000), followed by Papua New Guinea (29.59 DALYs per 100,000; 95% UI: 7.14 to 73.73 DALYs per 100,000), Burkina Faso (25.48 DALYs per 100,000; 95% UI: 3.86 to 106.98 DALYs per 100,000), Yemen (25.37 DALYs per 100,000; 95% UI: 8.87 to 89.1 DALYs per 100,000), and Chad (24.93 DALYs per 100,000; 95% UI: 4.33 to 93.93 DALYs per 100,000; Fig. 1).

The lowest burden of OFC is in Canada (0.55 DALYs per 100,000; 95% UI: 0.34 to 0.86 DALYs per 100,000), Spain (0.9 DALYs per 100,000; 95% UI: 0.56 to 1.36 DALYs per 100,000), Greenland (0.9 DALYs per 100,000; 95% UI: 0.56 to 1.36 DALYs per 100,000), Uruguay (0.94 DALYs per 100,000; 95% UI: 0.59 to 1.44 DALYs per 100,000), and Portugal (0.95 DALYs per 100,000; 95% UI: 0.59 to 1.47 DALYs per 100,000).

The countries with the highest prevalence of OFC are Palestine (149.05 per 100,000; 95% UI: 120.96 to 178.56 per 100,000), Qatar (140.36 per 100,000; 95% UI: 111.3 to 174.03 per 100,000), Pakistan (138.5 per 100,000; 95% UI: 109.58 to 171.91 per 100,000), Bangladesh (114.29 per 100,000; 95% UI: 89.65 to 143.66 per 100,000), and Bhutan (113.93 per 100,000; 95% UI: 89.43 to 142.29 per 100,000).

The lowest prevalence is in Canada (8.47 per 100,000; 95% UI: 6.67 to 10.37 per 100,000), Greenland (13.03 per 100,000; 95% UI: 10.21 to 16.02 per 100,000), Spain (13.37 per 100,000; 95% UI: 10.75 to 16.27 per 100,000), Uruguay (13.87 per 100,000; 95% UI: 10.86 to 17.26 per 100,000), and Portugal (14.6 per 100,000; 95% UI: 11.37 to 17.98 per 100,000).

### 3.4. Global trends and projections

**Figure 2.**
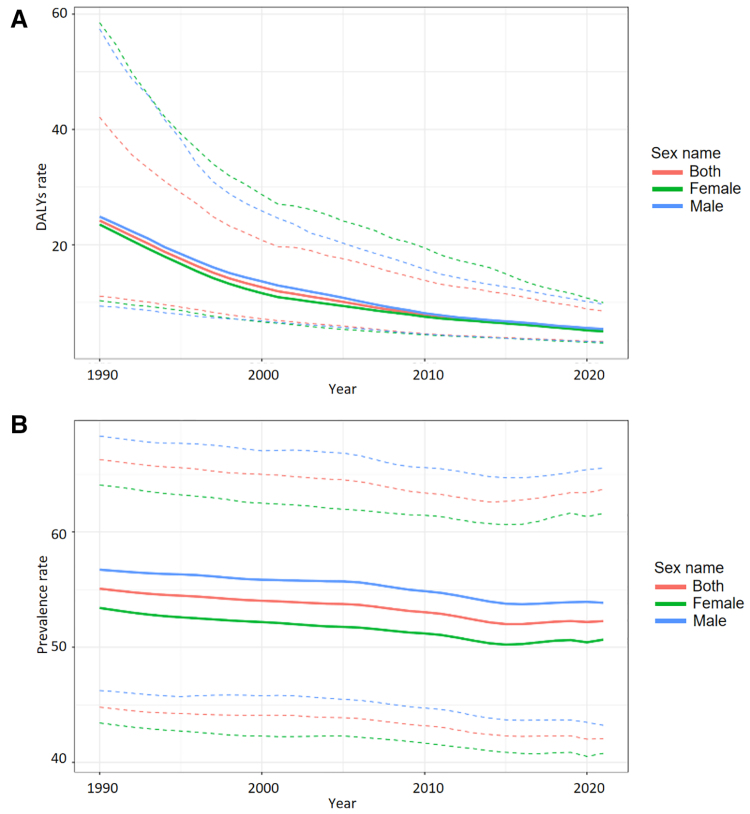
Trends in disability-adjusted life years (DALYs) rate (A) and prevalence rate (B; per 100,000 population) of orofacial clefts by gender globally from 1990 to 2021.

From 1990 to 2021, the DALY rate and prevalent rate of OFC showed an overall declining trend, with change rates of −5.12% (95% UI: −9.52% to −0.9%) and −78.59% (95% UI: −85.98% to −63.85%) respectively. Gender-specific analysis revealed that over the past 30 years, the DALY rate and prevalent rate of OFC in males have been higher than in females, but the gap in DALY rates between the sexes has gradually narrowed (Fig. 2).

**Figure 3.**
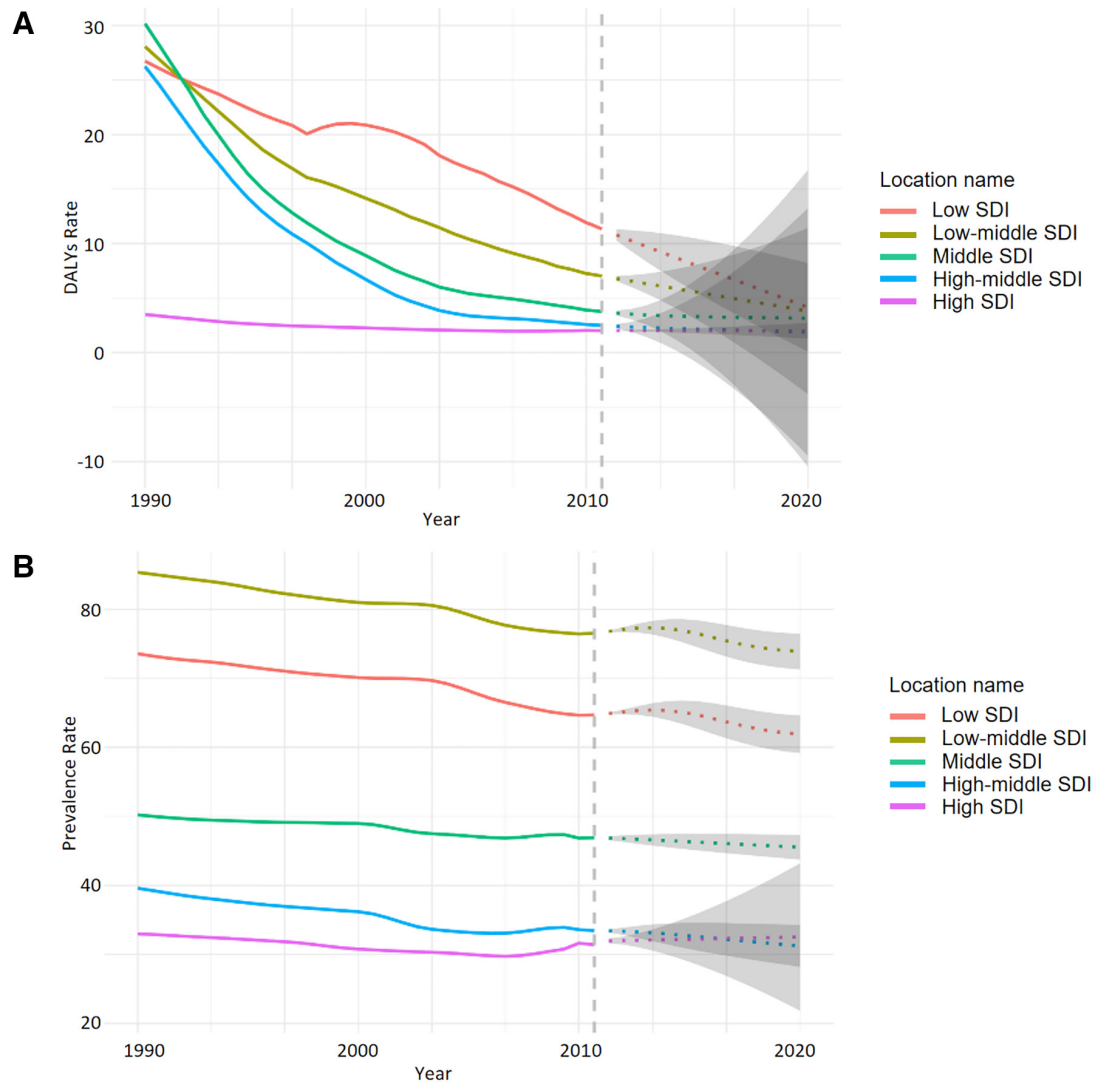
Trends and projections of disability-adjusted life years (DALYs) rate (A) and prevalence rate (B; per 100,000 population) of orofacial clefts in different SDI regions from 1990 to 2035. SDI = Socio-demographic Index.

It is projected that over the next 10 years, the global DALY rate and prevalent rate of OFC will continue to show a yearly decline. However, in high-SDI regions, the decline will be less significant, showing a more stable trend (Fig. 3).

## 4. Discussion

Using GBD 2021, this study characterizes the global distribution and burden of OFCs and shows substantial heterogeneity by region and socioeconomic context. Burden estimates are consistently higher in low-SDI settings than in middle- to high-SDI settings, underscoring persistent inequities in prevention, access to repair, and longitudinal care.

Firstly, the global burden of OFC is especially severe in low-SDI regions, mainly due to a lack of medical resources, inadequate basic health infrastructure, and insufficient awareness and intervention regarding congenital defect diseases in these areas. These regions often lack trained healthcare professionals who can provide high-quality surgical treatment, leading to many patients not receiving timely surgical repair or only receiving basic, inadequate treatment.^[12–14]^ The results show that Oceania, Western sub-Saharan Africa, and South Asia have the highest burden of OFC. In these low-income countries, social prejudice and stigma against individuals with OFC are particularly severe, exacerbating patients’ psychological distress and social isolation.^[15–17]^ Consequently, patients not only face significant physical challenges, such as feeding, speech, and breathing issues, but also bear a substantial psychological burden, including feelings of inferiority, anxiety, and depression.^[18]^ The combined effects of these factors further reduce patients’ quality of life and increase the economic burden on families and society.^[19]^

Secondly, although the burden of OFCs is relatively lower in high-SDI regions, important gaps remain. In high-income settings such as North America and Western Europe, access to surgical repair and multidisciplinary care is generally strong, and long-term outcomes are favorable.^[20]^ However, resources are unevenly distributed and social support can be thin. People in remote areas or with lower incomes may still struggle to obtain timely, comprehensive, and sustained multidisciplinary care. Moreover, after successful surgery many patients continue to face speech problems, dental and orthodontic needs, and psychosocial distress, underscoring the need to optimize resource allocation, strengthen community-based services, and ensure continuity of care.^[21]^

Additionally, the study shows a negative correlation between SDI and the burden of OFC, reflecting the significant impact of socioeconomic conditions on disease management. Low-SDI countries often lack comprehensive healthcare infrastructure and public health systems capable of effectively addressing the complex challenges posed by congenital diseases.^[22,23]^ Meanwhile, deficiencies in education and public health campaigns in these countries also limit public awareness of OFC, further restricting early diagnosis and timely treatment.^[24,25]^ In contrast, higher-SDI countries are equipped with better healthcare conditions and social support systems, allowing for multidisciplinary integrated treatment that includes surgical repair, speech therapy, psychological support, and rehabilitative care.^[26]^ These factors collectively contribute to significantly reducing patients’ disease burden and improving their quality of life.

Moreover, the burden extends beyond physical morbidity to pronounced psychological and social consequences.^[27,28]^ Visible facial differences expose children in particular to ridicule and exclusion, with lasting effects on mental health. Delayed or inadequate repair is associated with higher risks of psychological trauma, anxiety, and depression.^[29,30]^ Future strategies should therefore couple improved access and quality of surgery with structured psychological care and social-integration programs for patients and families to counter stigma-related harm.

This study has several limitations. First, GBD estimates depend on the availability and quality of underlying data sources, and under-ascertainment or misclassification may occur in settings with incomplete surveillance, limited registry coverage, or constrained vital statistics systems – particularly in low-SDI regions. Second, the GBD modeling framework synthesizes heterogeneous inputs using standardized methods; although UIs are provided, residual bias may persist due to differences in case definitions, diagnostic practices, and reporting over time and across countries. Third, our ARIMA projections are scenario-based forecasts that assume continuation of historical patterns; future changes in surgical capacity, access to multidisciplinary care, prevention strategies, and health policy may alter trajectories, and long-horizon predictions should therefore be interpreted cautiously.

Finally, these findings underline the role of global health policy in reducing the OFC burden. Priorities for low-SDI regions include strengthened international collaboration, targeted financing, and technical assistance to expand surgical capacity and comprehensive care. Public-health education is essential: broader awareness can reduce stigma, promote early presentation and treatment, and improve quality of life. Further research should test pragmatic clinical and social interventions across diverse socioeconomic settings to lessen the burden of OFCs and advance equity.

## 5. Conclusion

Based on GBD 2021 data,this study provides a comprehensive overview of the global burden and geographical distribution of OFC, along with their complex links to socio-demographic factors. The findings underscore the urgent need to strengthen healthcare access and service quality in low- and middle-income countries, where disparities remain most pronounced. At the same time, they highlight the importance of improving resource allocation and reinforcing social support systems within high-income settings to ensure continuity and equity of care.

## Acknowledgments

We thank all the individuals who contributed to the Global Burden of Disease Study 2021 for their extensive support in finding, cataloguing, and analyzing data and facilitating communication between and among team members.

## Author contributions

**Supervision:** Haoxuan Sun.

**Validation:** Weiling Chen.

**Writing – original draft:** Jiangbo Xin.

**Writing – review & editing:** Haoxuan Sun.
